# Metastatic and recurrent adrenocortical cancer is not defined by its genomic landscape

**DOI:** 10.1186/s12920-020-00809-7

**Published:** 2020-11-04

**Authors:** Tito Fojo, Lyn Huff, Thomas Litman, Kate Im, Maureen Edgerly, Jaydira del Rivero, Stefania Pittaluga, Maria Merino, Susan E. Bates, Michael Dean

**Affiliations:** 1 Columbia University Irving Medical Center, New York, NY 10032 USA; 2 James J. Peters Bronx VA Medical Center, Bronx, NY USA; 3 Laboratory of Cell Biology, Center for Cancer Research, National Cancer Institute, Bethesda, MD 20892 USA; 4 Department of Immunology and Microbiology, University of Copenhagen, Copenhagen, Denmark; 5 Laboratory of Translational Genomics, Division of Cancer Epidemiology and Genetics, National Cancer Institute, Bethesda, MD 20892 USA; 6 Center for Cancer Research, National Cancer Institute, Bethesda, MD 20892 USA; 7 Developmental Therapeutics Branch, Center for Cancer Research, National Cancer Institute, Bethesda, MD 20892 USA; 8 Laboratory of Pathology, Center for Cancer Research, National Cancer Institute, Bethesda, MD 20892 USA

**Keywords:** Adrenocortical cancer, Genomic landscape, Endocrine cancer, Adrenal cancer, Driver mutation

## Abstract

**Background:**

Adrenocortical carcinoma (ACC) is a rare, often-aggressive neoplasm of the adrenal cortex, with a 14–17 month median overall survival. We asked whether tumors from patients with advanced or metastatic ACC would offer clues as to putative genes that might have critical roles in disease progression or in more aggressive disease biology.

**Methods:**

We conducted comprehensive genomic and expression analyses of ACCs from 43 patients, 30 female, and 42 from metastatic sites, including deep sequencing, copy number analysis, mRNA expression and microRNA arrays.

**Results:**

Copy number gains and losses were similar to that previously reported for ACC. We identified a median mutation rate of 3.38 per megabase (Mb). The mutational signature was characterized by a predominance of C > T, C > A and T > C transitions. Only cancer genes *TP53* (26%) and beta-catenin (*CTNNB1*, 14%) were mutated in more than 10% of samples. The TCGA-identified putative cancer genes *MEN1* and *PRKAR1A* were found in low frequency—4.7 and 2.3%, respectively. The majority of the mutations were in genes not implicated in the etiology or maintenance of cancer. Specifically, amongst the 38 genes that were mutated in more than 9% of samples, only four were represented in Tier 1 of the 576 COSMIC Cancer Gene Census (CCGC). Thus, 82% of genes found to have mutations likely have no role in the etiology or biology of ACC; while the role of the other 18%, if any, remains to be proven. Finally, the transcript length for the 38 most frequently mutated genes in ACC is statistically longer than the average of all coding genes, raising the question of whether transcript length in part determined mutation probability.

**Conclusions:**

We conclude that the mutational and expression profiles of advanced and metastatic tumors are very similar to those from newly diagnosed patients—with very little in the way of genomic aberration to explain differences in biology. With relatively low mutation rates, few major oncogenic drivers, and loss of function mutations in several epigenetic regulators, an epigenetic basis for ACC may be postulated and serve as the basis for future studies.

## Background

Adrenocortical carcinoma (ACC) is a rare, often aggressive, and frequently fatal neoplasm of the adrenal cortex. A report describing trends in ACC in the United States found an age adjusted annual incidence of ~ 1/1,000,000 population with the incidence largely unchanged since the mid 1970′s [1]. The median age was 55, with a female to male preponderance of 1.34:1, a Whites to non-Whites ratio of 6.3:1, and most still diagnosed in advanced stages [1]. The rarity of this malignancy creates problems for its detailed study, even in large academic centers. Epidemiologic studies outside the United States have also indicated the rarity of the disease, at 1/1 million in the Netherlands, although a cluster with an incidence of 3-4/1 million has been found in children in Brazil [2, 3].

Prognosis is poor, with a 14–17 month median overall survival, and 5-year mortality rates of 75–90% [1, 2, 4]. Multiple strategies have been utilized to identify prognostic factors, including clinical, transcriptomic, epigenomic, and genomic. A staging system by the European Network for the Study of Adrenal Tumors (ENS@T) based on a retrospective analysis of data from 416 patients in the German ACC registry utilizes clinical data [5]. Transcriptomic analyses initially identified two clusters indicating poor (C1A) and good (C1B) prognosis groups [6, 7]. de Reyniès et al. also found in 92 patients that “the combined expression of BUB1B and PINK1 was the best predictor of overall survival” [6]. A transcriptomic analysis utilizing The Cancer Genome Atlas (TCGA) dataset confirmed the C1A/C1B subgroups, refining the data into four subgroups based on expression of steroid differentiation genes and proliferation genes, with high steroid/high proliferation having the poorest outcome [8]. Others have reported that hypermethylation is associated with a poor survival [9] including in particular hypermethylation of the *G0S2* gene [10]. One study that examined 203 ACC tumors in a validation cohort concluded that CpG island methylation analysis was an independent prognostic marker of survival in ACC, “independent of the best established prognostic factors, including tumor stage and Ki67” [11].

Somatic mutations have also been examined as prognostic markers. Genomic studies reported *TP53* and *CTNNB1* (encoding beta-catenin) mutations to be mutually exclusive and found in patients with ACC who had a “poor outcome” [12, 13]. Both held up as poor prognostic markers in a later multivariate analysis [14]. Importantly all survival curves have late plateaus that demonstrate “prolonged survival in occasional patients” with metastatic disease, although the biology for this phenomenon is not understood. The best prediction models integrate multiple of the above noted features [15].

Two comprehensive genomic analyses including the TCGA dataset identified alterations in *CTNNB1*, *TP53*, *CDKN2A*, *RB1* and *MEN1*—genes that had previously been reported as mutated in ACC—as well as in *ZNRF3*, *DAXX*, *TERT* and *MED12* [8, 16]. Collectively, alterations in *ZNRF3, CTNNB1, APC* and *MEN1* suggested that the Wnt/beta-catenin pathway could be a common pathway involved in ACC carcinogenesis. And while the authors reported numerous mutations and DNA methylation alterations in ACCs with poor outcome and specific deregulation of two microRNA clusters in ACCs with good prognosis, it was unclear how these changes could translate into potentially “druggable” findings. A recent review suggested as many as 10% of mutations could be actionable [17]. A recent genomic analysis of metastatic disease concluded there was a higher mutation rate but without a common mutation profile emerging [18].

In the present study, we report on a distinct group of ACCs, with disseminated metastatic disease. We began this effort hoping to discern potential biologic underpinnings for bad outcomes. We conclude that ACC remains very much a mystery—with advanced and metastatic tumors very similar to those from newly diagnosed patients—with very little in the way of genomic aberrations to explain it.

## Methods

### Sample collection, DNA, and RNA isolation

The tumors analyzed for these studies were obtained from patients with ACC seen at the National Cancer Institute, National Institutes of Health (NIH) in Bethesda, Maryland, USA from 1995 to 2015, a period of time when over 300 patients were evaluated and treated at the center. Tumor samples were collected at the time of surgical resection of sites of recurrence and in one instance extensive locally advanced disease; no primary tumors are included in this patient cohort. When possible, a paired normal buffy coat was obtained for each patient. Normal adrenal tissue was obtained from the Cooperative Human Tissue Network (CHTN), Southern Division, Birmingham, AL. The H295 cell line was obtained from ATCC (Manassas, VA) [19]. Ethics approval for the analyses reported here was provided by the Institutional Review Board (IRB) of the National Cancer Institute in Bethesda, MD, USA. Samples were obtained from patients distinct from those reported by Gara et al. [18].

Tumor samples were fresh frozen on dry ice, embedded in OCT, sectioned, stained with hematoxylin and eosin and reviewed by a pathologist (MM and SP). DNA for Exome Sequencing was isolated using the Qiagen DNA micro Kit (Qiagen, Valencia, CA). DNA was extracted only on tissue sections that were ≥ 75–80% tumor. DNA extraction was also performed on 1–5 × 10^6^ cells obtained from patient buffy coat samples. RNA was extracted from 50–100 mg of patient tumor using the Qiagen miRNeasy Kit (Qiagen, Valencia, CA). Extracted RNA was then used for cDNA and miRNA array analyses.

### Exome capture, sequencing and analysis

Genomic DNA (3 µg, quantitated by fluorometer and agarose gel) from 43 adrenal tumors, 25 buffy coats and the H295R cell line was isolated, fragmented and exome captured.

Exome target capture and enrichment were performed using Agilent SureSelect Human All Exon V4 target enrichment kit (Agilent Technologies, Santa Clara, CA), which targeted 51 megabases of sequence (20,965 genes, and 334,378 exons and miRNAs). Exome libraries were prepared by following manufacturer’s recommended protocols. Sequencing was performed using an Illumina HiSeq2000 (Illumina, San Diego, CA), the sequencing was run as 2 × 100 base pairs with TruSeq V3-HS reagents. The HiSeq Real Time Analysis (RTA 1.12.4.2) was used for processing image files, and the Illumina CASAVA 1.8.2 was used to demultiplex and convert binary base calls and qualities to fastq format. The sequence reads were aligned to human genome (GRCh37/USCS hg19) reference using the CASAVA 1.8.2 alignment module (ELANDv2e gapped alignment). SNPs and Indels were called using CASAVA 1.8.2 variant detection module. Variants were annotated using ANNOVAR [20]. Variants covered by less than 10 high quality reads were excluded. Variants in segmental duplications and known repeat regions were removed, as these are likely false positives. This study aimed to focus on somatic genetic changes, therefore identical variants observed in > 4 samples and those observed in > 1% of the 1000 Genomes Project [21] or NHLBI GO Exome Sequencing Project (ESP6500) [22] were also removed as likely germline variants. Additionally, variants in olfactory receptor genes were also not included in analyses. Whole Genomic Sequencing fastq files were deposited in Sequence Read Archive (SRA); the link for the Fastq files can be found in SRA: https://trace.ncbi.nlm.nih.gov/Traces/study1/?acc=PRJNA596175

Mutation signatures were analyzed by the MutaGene program [23, 24]. Separate analyses of tumor only and tumor normal (blood DNA) pair data gave similar results. Mutation profiles were compared to two other ACC studies with filtering as follows: COMETE (n = 45, cutoff = 4) [16], TCGA (n = 91, cutoff = 9) [8], and NIH (n = 43, cutoff = 7).

### Variant validation

Selected variants identified in tumor exomes were validated by manual review using IGV software (Broad Institute) [25, 26]. When variant frequency was low, primers were designed using Primer 3 [https://biotools.umassmed.edu/bioapps/primer3_www.cg] and synthesized by Life Technologies (Carlsbad, CA). Phusion Hi Fidelity PCR reagents (NE Biolabs, Ipswich, MA) and Eppendorf Gradient thermal cycler (Hauppauge, NY) were used to generate 200 bp amplicons that were validated by agarose gel electrophoresis. Big Dye v.1.1 chemistry (ABI, Foster City, CA) sequencing was performed on each amplicon and then subjected to chromatography on a 3130XL Genetic Analyzer (ABI). Chromatograms were examined visually to confirm called variants.

### Copy number analysis

Copy number was derived from the exome sequence of 25 pairs of matched tumor/normal (blood) samples using ngCGH [27]. Windows of 1000 reads in the normal sample were used to calculate a log2 ratio of tumor and normal reads and ratios were then median centered. These ratios were imported into Nexus 7.5 (Biodiscovery, Hawthorne, CA) and the Fast Adaptive States Segmentation Technique (FASST2) was used to make CNV calls. A significance threshold of 5e^−6^ was used to adjust the sensitivity of the algorithm and a minimum number of 20 amplicons per segment were used to eliminate small CNV segments. 0.2/-0.2 were used as cutoffs for gain/loss and 0.6/-1.0 for high gain/high loss. Eight tumors were removed from analysis due to excessive noise. Regions significantly altered in the remaining 17 tumors (as compared to their paired normal sample) were defined and gene ontology (GO) terms over-represented in these regions were identified.

### Gene expression by microarray profiling

Global gene expression analysis was performed according to protocol using the Affymetrix PrimeView platform, which was applied on 63 samples including 43 patients (some with replicates), 5 normal adrenal tissue controls, and 1 ACC cell line (H295R). Gene expression data were summarized by the SST-RMA (signal space transformation—robust multi-array average) gene level method implemented in the Transcriptome Analysis Console (TAC) 4.0 software (Thermo Fisher Scientific, Waltham, MA). Differentially expressed genes (DEG) were identified by comparing the ACC group to the group of 5 normal controls (Welch t-test, cut-off: twofold change and P < 0.05). Significance was adjusted for multiple testing by estimating false discovery rates (FDR)^1^. Data were visualized in Qlucore Omics Explorer v. 3.4 (Qlucore AB, Lund, Sweden), including principal component analysis (PCA), heat maps, and unsupervised hierarchical clustering. Functional analysis, including pathway, upstream regulator, and network analysis, was performed in Ingenuity Pathway Analysis (IPA, Qiagen, Redwood City, CA). The expression data are deposited in Gene Expression Omnibus (GEO), accession number *GSE143383* [28].

### MicroRNA array profiling

The RNA quality was verified by an Agilent 2100 Bioanalyzer profile, and 450 ng total RNA from sample and a common reference pool was labelled with Hy3™ and Hy5™ fluorescent label, respectively, using the miRCURY™ LNA Array power labelling kit (Exiqon, Denmark) following the procedure described by the manufacturer. The labeled RNA samples were hybridized to the LNA-enhanced miRCURY™ microarray version 11.0 (Exiqon, Denmark), which contained capture probes targeting all miRNAs for human, mouse or rat registered in the miRBASE version 13.0 at the Sanger Institute at the time of the analysis. The hybridization was performed according to the miRCURY™ LNA array manual using a Tecan HS4800 hybridization station (Tecan, Austria). After hybridization, washing and drying, the microarray slides were scanned in an Agilent G2565BA Microarray Scanner System (Agilent Technologies, Inc., USA) and the resulting images were quantified by ImaGene v.8.0 software (BioDiscovery, Inc., USA). The quantified signals were background corrected (Normexp with offset value 10)^3^ and normalized using the global LOWESS (LOcally WEighted Scatterplot Smoothing) regression algorithm. The miRNA expression data are deposited in Gene Expression Omnibus (GEO) accession number *GSE143385* [28].

## Results

We performed genomic analysis on a set of 69 samples that included (1) tumor tissue from 43 patients with ACC who underwent resection of metastatic disease (one patient #44 had locally advanced disease with extension into surrounding tissue); (2) matching normal DNA from 25 of the 43 patients; and (3) the ACC cell line, H-295, established from a carcinoma of the adrenal cortex [19] (Table 1). Thirty of the forty-three were women. Median age of females was 46; median age of males was 52. Patients were likely to have had prior systemic chemotherapy and even refractory disease. We utilized a combination of approaches, including exome sequencing, mRNA expression arrays and miRNA expression arrays. We performed exome capture followed by paired-end sequencing on the 69 samples itemized above. The mean depth of coverage was 115X per sample, with an average of 98% of targeted bases covered by ≥ 10 reads (Additional file 1: Figs. 1A and 1B).

**Fig. 1:**
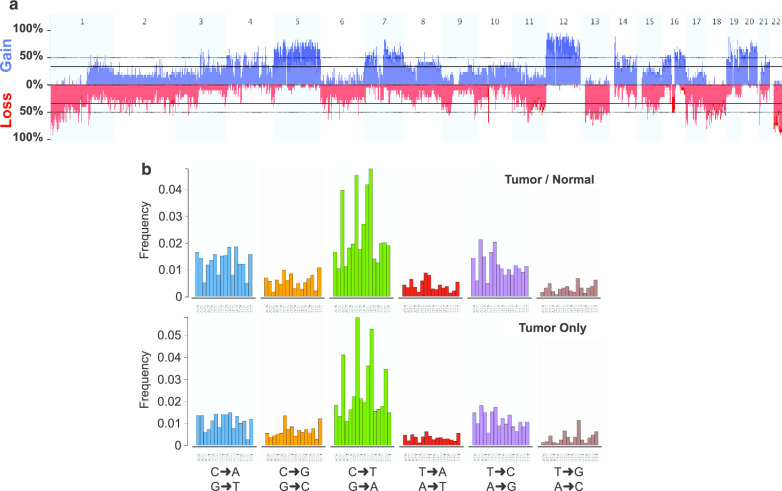
**a** Frequency of copy number gains/losses in 17 of the 25 ACC tumors with paired normal DNA. The y-axis represents the percentage of samples with gain/loss.[ngCGH + Nexus segmentation]. Eight tumors removed due to excess noise. **b** Mutational Signature in 25 samples from Tumor/Normal pairs and in 18 samples with Tumor Only analysis

**Table 1:** Demographics for patients whose tumors were subjected to whole exome sequencing (n = 43)

MS ID**	Sex	Age***	Mitotane	Systemic therapy prior to metastatectomy	DOD	Hormonally active
13	F	20′s	Yes	Mitotane MAVE Taxol Cisplatin	*Alive*	Yes, Cushing syndrome
43	F	20′s	Yes		Dead	Yes, Cushing syndrome
9	F	20′s	Yes		*Alive*	Unknown
17	F	20′s	Yes		Dead	No
4	F	20′s	No		Dead	Yes, Cushing syndrome
30	F	20′s	Yes	Mitotane	*Alive*	No
32	F	30′s	Yes	T-MAVE	Dead	No
11	F	30′s	Yes	EDP	*Alive*	No
38	F	30′s	Yes		*Alive*	Yes, Cushing syndrome
12	F	30′s	Yes	T-MAVE	*Alive*	No
31	F	40′s	Yes	T-MAVE	Dead	No
36	F	40′s	Yes		Dead	Yes, aldosterone
26	F	40′s	Yes	MAVE	Dead	
42	F	40′s	Yes		Dead	Yes, testosterone
3	F	40′s	Yes	T-MAVE Gemzar/Cisplatin	*Alive*	Yes, based on symptoms
34	F	40′s	No		Dead	Yes, Cushing syndrome
40	F	40′s	Yes	Mitotane × 2 months	Dead	No
1	F	40′s	Yes	EDP	Dead	Yes, based on symptoms
27	F	40′s	No	Cisplatin prior to primary resection; cisplat in & axitinib prior to metastatic resection	Dead	Yes, Cushing syndrome
23	F	50′s	Yes		Dead	Yes, likely Cushing syndrome
2	F	50′s	Yes	IMC-A12, mitotane	Dead	Yes, Cushing syndrome
16	F	50′s	Yes	T-MAVE	Dead	Yes, Cushing syndrome
44	F	50′s	Yes	EDP/M	*Alive*	Yes, Cushing syndrome based on symptoms
15	F	50′s	Yes	EDP/M	Dead	No, palpitations, HTN, sweating
25	F	50′s	Yes	Mitotane	*Alive*	No
21	F	50′s	Yes	OSI-906, EDP/M, Streptozocin	Dead	No
29	F	50′s	Yes		Dead	Yes, based on symptoms
24	F	50′s	No		Dead	No, h/o HTN
5	F	50′s	Yes	EDP/M, Streptozocin	Dead	No
7	F	60′s	Yes	T-MAVE	*Alive*	No
39	M	30′s	No	PSC-Velban	*Alive*	No
35	M	30′s	Yes	MAVE	Dead	No
19	M	30′s	Yes	Mitotane	Dead	No
33	M	30′s	Yes	EDP/M	Dead	No, h/o hypokalemia
10	M	40′s	Yes	Mitotane, streptozocin, cisplatin	Dead	Yes, Cushing syndrome
8	M	50′s	No		Dead	No
37	M	50′s	Yes	Mitotane	Dead	Yes, Cushing syndrome
6	M	50′s	No		Dead	No
28	M	50′s	No	Cisplatin, mithramycin	Dead	Yes, estradiol
22	M	50′s	Yes	EDP/M	Dead	No
14	M	50′s	Yes	EDP/M	Dead	No
41	M	60′s	Yes	MAVE, Etoposide + Carboplatin	Dead	No
20	M	70′s	No		Dead	No

All samples except #44 were obtained from metastatic disease sites; #44 was locally advanced disease; #18 is omitted in the sequence

Abbreviations: MAVE = mitotane + Adriamycin (doxorubicin) + vincristine + etoposide; T-MAVE = tariquidar + MAVE; EDP = etoposide + doxorubicin + cisplatin;

**MS ID, Manuscript ID number

***Age range provided for privacy compliance

We used the exome sequence data to perform copy number analysis on the 25 tumors with matched normal samples using ngCGH [20] and Nexus segmentation (Biodiscovery, Hawthorne, CA). Eight tumors with excess noise were removed from further analysis. In the remaining 17 samples, significant gains were found on chromosomes 4, 5, 7, 8, 12, 14, 16, 19 and 20; with significant losses observed on chromosomes 1, 2, 6, 9, 10, 11,13, 15, 17, 18, 21 and 22 (GISTIC, *q* value < 0.05; Fig. 1a). In all, 55% of the genome was affected by copy number alterations. The copy number profiles of the tumors described here are very similar to Assie et al. and to the metastatic ACCs described by Gara et al. In all three studies there is a high proportion of tumors with a gain of all or nearly all of chr. 5, 7, 12, 19 and 20, and loss of chr. 1, 2, 6, 11, 13, 17, 18, and 22 [16, 18] (Additional file 1: Fig. 2A and B). By contrast, several of these chromosome alterations were rarely observed in the tumors of Zheng et al. [8]. We also found amplification of the *TERT* (5p15.33) and *CDK4* loci (12q14) in 76.5% and 82% of the tumor samples assessed, respectively; and deletions of the *CDKN2A* (9p21.3), *RB1* (13q14) and *ZNRF3* loci (22q12.1) in 35%, 59% and 76.5% of tumors, with the incidence of each higher than that previously reported [8, 16].

**Fig. 2:**
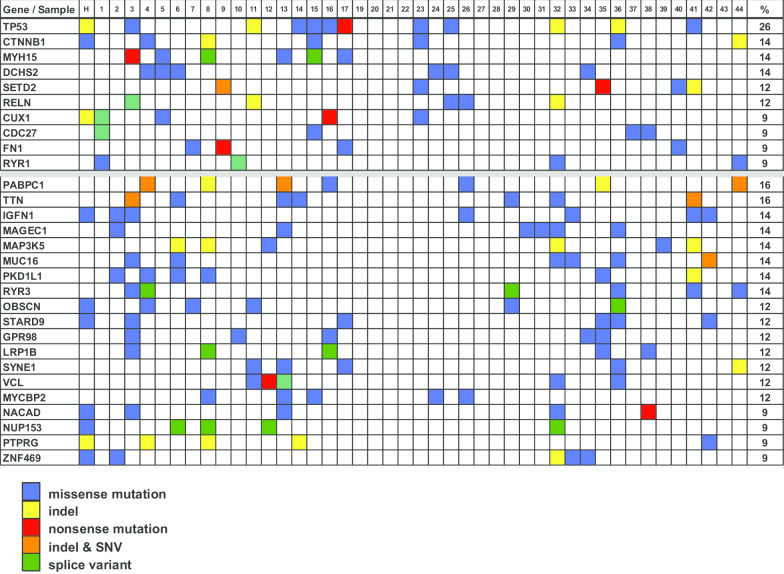
Mutations in individual patients. Genes that were mutated in $\ge$ 4 patients ($\ge$ 9%) and validated by a second sequencing method are shown. Column labeled H, depicts the results in the H295 cell line. Top portion: Mutations found in $\ge$ 9% of those samples with buffy coats; Lower portion: Mutations found in $\ge$ 9% of all samples. The last column indicates the percent mutated in the entire dataset

### Whole exome sequencing

After filtering using public databases and normal samples to remove likely false positive and germline variants and including only variants (and targeted bases) in exons or within 3 bp of exons (see Methods), we identified 4981 putative somatic mutations in 3814 genes in the 43 ACC samples, with a median mutation rate of 3.38 per megabase (Mb) in exonic + 3 bases (range 0.50—18). As regards the DNA mutation spectrum, it was characterized by a predominance of C > T, C > A and T > C transitions, with the 25 tumors with matched normal samples shown in the upper panel (Fig. 1b). Twenty-nine genes with non-silent alterations (missense, nonsense, insertions and deletions [indels] and splice variants within 3 bp of exon) present in > 9% of our cohort ($\ge$ 4/43) were each validated by manual inspection in the Integrated Genome Viewer [14, 15] and by PCR and Sanger sequencing. Figure 2 shows these mutations, with the upper set identified in at least 9% of patients in whom we had germline DNA, and the lower set the mutations identified in at least 9% of the whole population. The two most frequently altered genes, *TP53* and β-catenin (*CTNNB1*) were altered in 11 (26%) and 6 (14%) samples, respectively. Nine of the twelve *TP53* mutations (eleven patients) had variant frequencies of 70–90% suggesting LOH. *TP53* mutations preferentially affected the DNA-binding domain (10/12—83.3%) and one occurred in the transactivation 2 domain (1/12—8.3%) (Additional file 2: Table 1). By comparison, the *CTNNB1* mutations all occurred in the GSK-3B phosphorylation domain of the gene, a serine/tyrosine rich region.

**Fig. 3:**
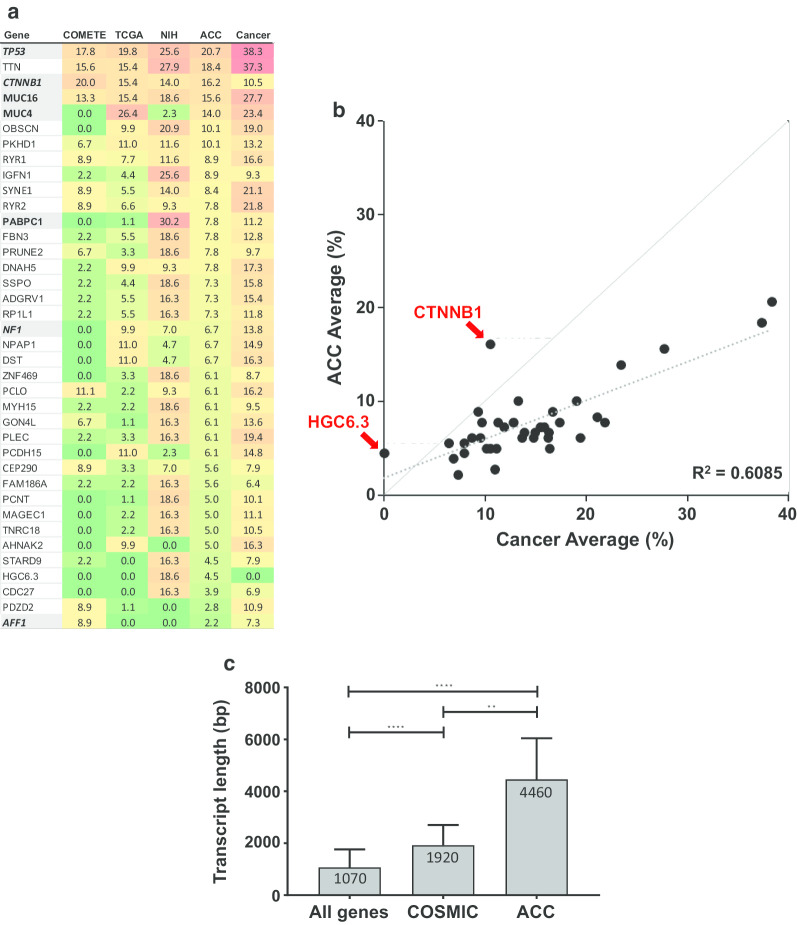
**a** ACC mutation frequencies. The heat map lists the frequencies of the 38 most commonly mutated genes across 3 ACC studies: COMETE (n = 45, cutoff = 4) [16], TCGA (n = 91, cutoff = 9) [8], and NIH (n = 43, cutoff = 7 [the current study]), as well as the average percentage of the three studies (ACC column, n = 179), and the cancer average as recorded in the TCGA PanCancerAtlas, which includes 33 cancers and 11.315 cases [https://gdc.cancer.gov/]. The heat map is sorted according to the average percentage of the three studies – the ACC column (i.e. number of patients with the mutation / total number of patients). The four genes shown in ***italic bold*** on shaded background (***TP53, CTNNB1, NF1 and AFF1***) are included in the 576 COSMIC Cancer Gene Census (CGC) Tier 1 genes, while the three genes in **bold** also on a shaded background (***MUC16, MUC4 and PABPC1***) are among the 147 CGC Tier 2 genes [REF.: COSMIC, Catalogue of Somatic Mutations in Cancer, https://cancer.sanger.ac.uk/cosmic]. **b** Correlation between the mutation frequency of the 38 cancer genes in 3A in ACC and in cancer in general both plotted as the average of their occurrence in ACC (Y-axis) and all cancers (X-axis). The diagonal identity line and the dashed linear regression line (R = 0.78, p = 1.6 E-08) are shown. The average mutation occurrence in ACC for 36 of the 38 genes, including TP53, fall below the identity line, indicating the mutation frequency is lower in ACC compared to the average in all cancers. The two exceptions are CTNNB1 and HGC6.3. **c** Median transcript length: comparing ACC (from 3 datasets) to all cancers in TCGA to all coding genes. Bar graph showing differences in median transcript length; comparing all coding genes from Ensembl Biomart (n = 37,416), the Tier 1 cancer-related genes in the COSMIC database (n = 576), and the most frequently mutated genes in the three ACC datasets (n = 38). The median with interquartile range is shown for each dataset. The transcript median length was statistically different between the 3 datasets: all coding genes vs. COSMIC (p < 0.0001); all coding genes vs. ACC (p < 0.0001); COSMIC vs. ACC (p < 0.01). The non-parametric Kruskal–Wallis statistic was applied for comparison of median values between the three groups

Next, we compared our data with two prior sequencing studies performed in ACC, the COMETE and TCGA-ACC datasets and with the multiple cancer types of the TCGA PanCancer Atlas (Fig. 3a) [8, 16, 29]. We found that the majority of genes in ACC that had mutations also have been found to have mutations in all cancers, suggesting that they were just as likely to have been found to harbor mutations in other cancers as in ACC. Four genes shown in ***italic bold*** on shaded background (***TP53, CTNNB1, NF1 and AFF1***) are included in Tier 1 of the 576 COSMIC cancer gene census (CGC), while three genes in **bold** but without italics also on a shaded background (***MUC16*, *MUC4 and PABPC1***) are among the 147 CGC Tier 2 genes [COSMIC]. For only two of the 38 genes were mutation rates higher than those in all cancers. These two, *CTNNB1* and *HGC6*, seem unique to ACC. Removing these two from the comparison, the regression coefficient for the incidence of the mutations in ACC and the incidence in all other cancers increased to R = 0.87, R^2^ = 0.76 (Fig. 3b). Finally, we compared transcript length for the 38 most frequently mutated genes in the 3 ACC datasets with the transcript length for the average of all coding genes, and with the transcript length for the COSMIC cancer census genes (Fig. 3c). We found a statistically significant difference between the three datasets with the transcript length of genes mutated in ACC being the longest. This suggested that at least some of the mutations, such as those in *TTN*, may have occurred as random events.

A recent analysis of TCGA data identified 300 **putative** cancer genes; including five identified as putative oncogenic drivers in ACC [8, 30]. Our analysis finds a similar higher incidence for the two most frequently mutated genes, *TP53* and *CTNNB1* (26% and 14%, respectively), as well as comparably low frequencies for the other three putative drivers—*ATRX*, 7.0%; *MEN1*, 4.7%; and *PRKAR1A*, 2.3%.

The p53-Rb and WNT-CTNNB1 pathways were most frequently altered. Inactivating mutations or homozygous deletions were found in *TP53* (11/43, 26%); no other p53-Rb pathway mutations were found, including in *CDKN2A*, *RB1, CDK4* or *MDM2*. In addition to the *CTNNB1* alterations (6/43, 14%) described above, mutations in the WNT-CTNNB1 pathway also included APC (2/43), AXIN1 (2/43), and AXIN2 (1/43) [31]. No mutations were detected in *GSK3B*, *CK1* or *TCF*/*LEF*. In all, this pathway appeared to carry mutations in 10/43 (23%) tumors from individual patients.

As more tumors have been subjected to sequence analysis the prevalence of mutation in genes encoding proteins regulating chromatin and mediating DNA damage and its repair has been recognized and in some cases associated with oncogenesis [32, 33]. We examined the occurrence of mutations in genes encoding epigenetic regulators and DNA repair pathways in the 43 patients. Among epigenetic regulators we found 6 (14%) with predicted loss of function mutations, and 24 (56%) unique tumors with any alteration in any epigenetic regulators (Additional file 2: Table 2A). Among those genes known to be involved in mismatch repair deficiency (MMR), we found mutations in seven (16%) tumors, including one with clear loss of function and 2 with loss of heterozygosity (Additional file 2: Table 2B). Among other types of DNA repair proteins, including those involved in the homologous recombination pathways, we found 14 (33%) tumors with alterations in these genes, including 2 frameshift mutations with predicted loss of function in the *WRN* and *BLM* genes, and the rest nonsynonymous with unknown effect on function. These included mutations in *BRCA1*, *BRCA2*, *PALB2*, and *POLE*. Included amongst the variants in epigenetic regulators were two with *ATRX* mutations and six with *SETD2* mutations, both known to be involved in DNA repair and with mutations in both associated with high mutation burden. Aggregating *ATRX*, *SETD2*, and MMR, homologous recombination (HR), and other genes involved in DNA repair, but excluding *TP53*, we found 36 mutations in samples obtained from 21 (49%) of the 43 patients. Together, these results suggest that impaired DNA repair may be an important theme in these aggressive ACCs.

**Fig. 4:**
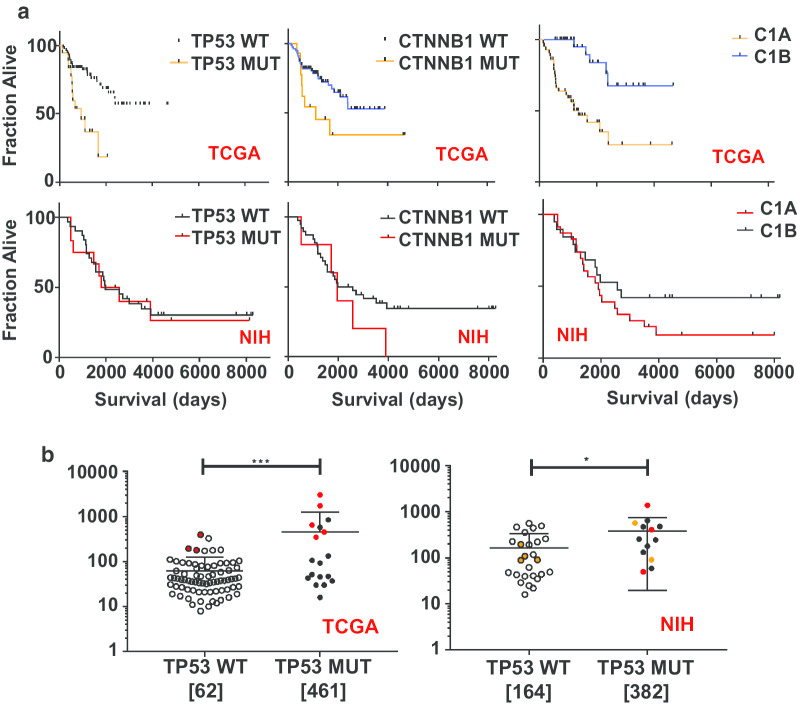
**a** Kaplan Meier plots evaluating the impact of *TP53* and *CTNNB1* mutation on overall survival in the TCGA and in the NIH datasets. These can be compared to the Kaplan Meier plots derived from gene expression array data for aggressive C1A and indolent C1B subtypes [6, 8]. **b** Total number of mutations for each subgroup are graphically depicted with the mean and SD. The numbers in brackets below each dotplot are the mean number of mutations for that subgroup. Mutation counts in the ACC TCGA dataset are higher in tumors harboring a TP53 MUT compared to those with TP53WT [***p = 0.001], although many of those patients were also noted to have mutations suggestive of MMR deficiency (red symbols). The correlation with TP53 MUT was weaker in the smaller NIH dataset [*p = 0.05]. Orange symbols indicate that the mutation counts were additionally filtered by germline sequencing in those patients. None of the patients in the NIH set had germline *TP53* mutations

The occurrence of *TP53* mutations in ACC was already well-known and considered a negative prognostic factor in adults and children [34, 35]. We asked whether *TP53* or $\beta$-catenin mutations had an impact on overall survival in either the NIH cohort, or in the TCGA cohort. In the Kaplan Meier plots shown in Fig. 4a, we demonstrate that, while *TP53* mutations are associated with worse outcome in the patients that comprise the TCGA data, *TP53* was not able to discriminate survival in the NIH cohort. Results with $\beta$-catenin were less definitive. Recently, the success of immunotherapy has led to development of biomarkers, including that of tumor mutational burden (TMB), which can be associated with better outcome following immunotherapy [36, 37]. We found occasional tumors with elevated TMB, and only a weak association between TMB and *TP53* mutation (Fig. 4b). While there was an association in the TCGA data, the results may be confounded by the presence of mutations suggestive of MMR deficiency.

### Gene expression arrays

**Fig. 5:**
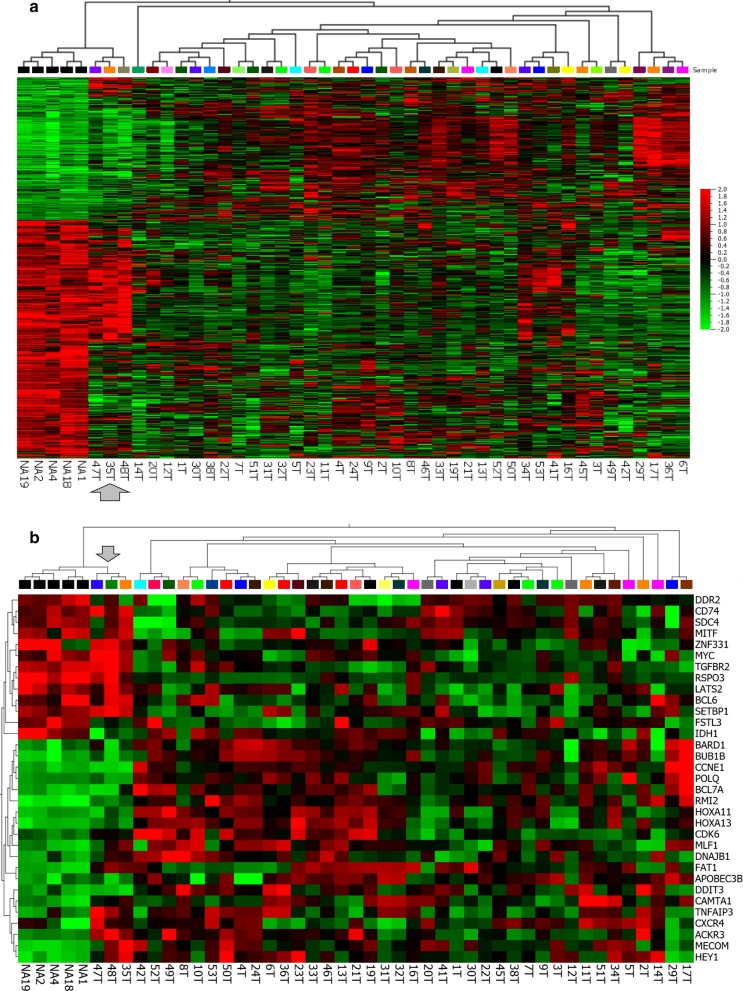
Results of cDNA Array analysis of ACC gene expression. **a** Heat-map and unsupervised hierarchical clustering based on the 769 genes that differ most between ACC and NA **(**$\sigma /\sigma \mathrm{max}$ >0.2, p < 0.05, q = 0.18, > 2-FC, one sample per patient, probe-sets collapsed to median per gene). Compared to the normal adrenal, 289 DEG were noted to be upregulated, and 480 downregulated in the tumors. The ACC samples cluster separately from the normal adrenal (NA) samples, with the exception of 35 T, 47 T, and 48 T (gray arrow) that cluster together with the NA samples (Note: as these tumor samples were from metastasectomies normal adrenal tissue could not have contaminated the samples). **b** Heat-map and unsupervised hierarchical clustering based on the 33 out of 576 Tier 1 COSMIC cancer census genes that differ the most between ACC and NA ($\sigma /\sigma \mathrm{max}$ >0.2, p < 0.05, q = 0.17, > 2-FC). The ACC samples cluster separately from the NA. Again, 35 T, 47 T, and 48 T cluster together with the NA samples

In addition to the genomic analysis we conducted a gene expression analysis in 57 tumor samples and five normal adrenal samples using the Affymetrix PrimeView arrays 49,395 probe sets. Figure 5a shows a heat-map and unsupervised hierarchical clustering reflecting the 769 differentially-expressed genes (DEGs) separating ACC and NA ($\sigma /\sigma \mathrm{max}$ >0.2, p < 0.05, q = 0.18, > 2-FC, one sample per patient, replicates removed). Most of the ACC samples clustered separately from the NA, but interestingly, three of the samples, 35 T, 47 T, and 48 T clustered together with the NA samples, suggesting they are more “normal-like”. Among the 769 DEGs, 289 were upregulated and 480 downregulated (Additional file 2: Table 3). Box plots demonstrated that there was a wide expression range in the ACC samples, relative to the normal adrenal (Additional file 1: Fig. 3A and 3B). Furthermore, in a heat-map and HCL plot of the 1666 most variable genes ($\sigma /\sigma \mathrm{max}$ >0.3) to look at how well different samples from the same patient segregated together, we analyzed 57 tumors samples, the H295 cell line and five normal adrenal samples [data not shown]. As in Fig. 5a, the five normal adrenal samples clustered together. For nine of the patients more than one metastatic tumor was analyzed and in seven of the nine cases the different tumor samples segregated next to each other in this unsupervised analysis including six different lung metastases from one patient all of which clustered together.

Additionally, we looked to see whether the DEGs were among the known cancer genes, using the Catalog of Somatic Mutations in Cancer (COSMIC) Cancer Gene Census (CGC) [38]. Out of 576 COSMIC Tier 1 genes, 40 (6.9%) met the significance criteria (fold change > 2.0 and P < 0.05) (Fig. 5b); and among 147 Tier 2 COSMIC genes, only 12 (8.2%) met the same significance criteria. As with the 769 DEGs, three ACC samples clustered with the normal adrenal samples. None of the cancer genes were up- or down-regulated in a predominant manner.

We also analyzed our data according to the previous prognostic signatures validated in ACC, using 136 “K4” genes identified by Zheng et al. in the TCGA analysis (Additional file 1: Fig. 4A and B) [8]. We found samples clustering into the two groups C1A and C1B, representing more aggressive and indolent cancers, respectively [6, 7]. And we found that samples clearly clustered based on the adrenal differentiation genes (steroid high vs. low) but less well based on proliferation, with 18 samples indeterminant. Based on these assignments, Kaplan–Meier plots were derived, as shown in Fig. 4a and Additional file 1: Fig. 5. Our data are consistent with the overall poor prognosis of metastatic ACC.

We also analyzed the data by IPA for alterations in upstream regulators in known cancer drivers (Additional file 2: Table 4). Interestingly, p21 and TP53 were among the regulators predicted to be downregulated; consistent with the known loss of function mutations in *TP53*. In contrast, cyclin D1, MYC and E2F, well-known cancer drivers, were found upregulated.

### MicroRNA Profiling

Finally, we analyzed the microRNA profile in 24 of the ACC samples, compared to normal adrenal, finding that 86 microRNA were significantly different between the two datasets (listed in in Additional file 2: Table 5). Interestingly, there was also a set of tumors that were “normal adrenal-like”, clustering with the normal adrenal samples (Additional file 1: Fig. 6). However, these did not show any overlap with the “normal adrenal-like” samples from the RNA expression array.

## Discussion

We report a cohort of patients with advanced/metastatic adrenocortical cancer (ACC) whose tumors have undergone genomic and expression analyses. Unlike the previous cohorts that included primarily tumors harvested at the time of an initial presentation [8, 13, 16], all of the patients included in this analysis presented with advanced/metastatic disease or developed metastases. Consequently, all of the samples were obtained from patients at the time of subsequent metastasectomies, part of an aggressive management strategy that our team adopted in the management of patients with ACC [39]. While the ability to undergo a metastatectomy could select patients with somewhat more indolent disease, and thus not perfectly reflect the spectrum of advanced ACC, our clinical experience was that even patients with aggressive metastatic lesions could benefit. We were interested in determining whether the tumors from these patients, enriched for metastatic capacity, would offer clues as to putative genes that might have critical roles in disease progression. Instead we found the mutational profiles of our samples similar to those previously published, with few known cancer genes mutated and only *TP53* (26%) and beta-catenin (*CTNNB1*, 14%) as putative cancer drivers mutated in more than 10% of samples.

As in the majority of cancers, we found copy number gains and losses in many of our samples. We observed a CNV profile that was very similar to that seen in the Assie et al. samples [16], and in metastatic tumors [18] but distinct from that of the TCGA reported in Zheng et al. [8]. This likely reflects the fact that our sample is almost exclusively composed of metastatic ACCs and the TCGA study comprised 60% stage I and II tumors [8, 16, 18] (Fig. 1 and Additional file 1: Fig. 2).

We also identified 4981 putative somatic mutations in 3814 genes with a median mutation rate of 3.38 per megabase (Mb) in exonic + 3 bases (range 0.50—18). These values are somewhat higher than those in a previous study that reported a mean somatic mutation rate in coding sequences of 0.60 mutations per Mb [16], likely due to the inclusion of some noncoding variants and possibly to the more advanced presentation of our patient population. Differences in the mutational burden amongst different clinical presentations have been previously reported in other tumors [40]. The mutational signature was characterized by a predominance of C > T with a lower frequency of C > A and T > C transitions. The major predicted signatures were the C > T at CpG sites associated with aging (SBS1), found in all cancers, and a mismatch repair deficiency signature (SBS6). But despite the number of mutations, as a whole, our data and that in the previous analyses finds that that rate of mutations in ACCs is similar to that in other cancers, indeed even lower (Fig. 3). There are no common mutations in genes that would suggest a personalized therapy that would benefit a meaningful percentage of patients. Although a TCGA analysis identified *MEN1* and *PRKAR1A* as putative cancer genes in ACC, the low frequency of mutations—4.7% and 2.3% for *MEN1* and *PRKAR1A*, respectively—render their importance in this disease at best marginal [30]. Furthermore, we found in the current and in the previous analyses that the majority of the mutations were in genes not implicated in the etiology or maintenance of cancer. Specifically, amongst the 38 genes that were mutated in more than 9% of samples in any of the three studies, only four of the 38 genes can be found in Tier 1 of the 576 COSMIC Cancer Gene Census (CCGC), with three amongst the 147 CCGC Tier 2 genes [38, 41]. Thus, 82% of the genes with mutations likely have no role in the etiology or biology of ACC; while the role of the other 18%, if any, remains to be proven. Finally, we found the transcript length for the 38 most frequently mutated genes in the 3 ACC datasets to be statistically longer than the transcript length for the average of all coding genes, and for the COSMIC cancer census genes, an observation that raises questions as to the importance of many of the mutations, and whether their occurrence reflects in part the length of their transcripts and hence the probability of incurring a mutation (Fig. 3c).

Twenty-three genes mutated in our cohort were validated and as previously reported, the two most frequently altered genes were *TP53* and *CTNNB1*, mutated in 26% and 14%, respectively. The data for *TP53* suggested LOH in the majority and while an incidence rate of 26% represents a substantial fraction, it is lower than the majority of other cancers, something of a surprise in a cancer for which *TP53* has been generally regarded as important—and is thought to be etiologic in children who inherit a mutant *TP53* [42, 43]. This is especially surprising as our subjects all had advanced or metastatic disease, and *TP53* mutations have been reported to be higher in more aggressive disease for several cancers including ACC [34, 35, 44]. Interestingly, our analyses in part supports this bias by demonstrating that while *TP53* mutations are associated with worse outcome in the patients that comprise the TCGA data, composed primarily of tumors at initial presentation, the same was not true in our patient population. While we lack evidence to explain this discrepancy one could postulate that the aggressive presentations/biology of tumors in our patients were driven by more than the mutation of a single gene, and instead had a much more diverse expression/mutational profile.

As regards *CTNNB1*, the very modest rate of 14% mutations in our cohort and 16% across all ACCs is notably higher than in many cancers and may suggest a putative role in a subset of ACCs. It is also noteworthy that expression of *CTNNB1* is high in the normal adrenal and comparably high in ACCs, but also high across all tumors, precluding higher expression as an auxiliary/alternate mechanism. Other than *TP53* and *CTNNB1*, the mutational analysis revealed a high 18.6% incidence of *HGC6.3* mutations in our cohort, notably higher than the 0% in the other ACC cohorts and in all cancers. The importance of this remains uncertain and we would note that, unlike *CTNNB1*, *HGC6.3* remains uncharacterized, and is not in the COSMIC Cancer Gene Census (CCGC) [45].

Sequence data can inform in two ways. If mutations in a given gene or pathway are seen in a large enough fraction of a given tumor histology, it brings attention to that gene/pathway as having a possible critical role in the disease under study—with *BCR-ABL* in CML and *BRAF* in melanoma the two best examples [46, 47]. Alternately, when employed in everyday clinical practice, the hope is that such information will identify potentially druggable targets—the rationale for a “precision oncology” strategy—now widely applicable but lacking rigid scientific support [48, 49]. With three large data sets comprising nearly 200 patients with ACC now available, the data are robust enough to have identified key drivers. However, our data and that in the previous cohorts cannot be interpreted as having identified genes of much import to ACC, certainly not critical in its etiology or maintenance, nor identified druggable targets. Indeed, none of the five genes identified in the TCGA analysis as ACC-associated—*TP53*, *CTNNB1*, *ATRX*, *MEN1*, and *PRKAR1A*—can be argued to be truly “druggable”.

The clinical presentation of these cancers is all too often characterized by a very aggressive clinical picture, comprising very large tumors; invasion and destruction of surrounding tissue; large vessel involvement; metastasis to liver, lungs and bones; and intractability to chemotherapy. These are biologic properties that argue against a simple mutation-driven model as the cause of this cancer [50]. Instead it argues for other etiologies and given the plethora of clinical properties, epigenetic alterations must be entertained as a viable alternative [51, 52]. While we recognize some may argue this could be mediated by mutations, and the TCGA analysis identified “chromatin histone modifiers as pathways/biological processes affected by associated consensus driver genes” in ACCs [30], this allegation needs stronger supporting data given the plethora of genes that are implicated in epigenetic modifications—a very large number that also allows for much redundancy of function. The totality of the data also argues strongly against the notion that a mutational analysis can identify critical targets that may be addressed medically. Success in the case of the latter implicates such mutated genes as critical for ACC. To think that many such “individual” critical genes exist ignores the reality that ACC has an incidence of ~ 1 per million and cannot have a myriad of etiologies. Thus, these data argue against giving patients with ACC false hope that a “precision oncology” approach will identify what makes their tumor unique and bring any meaningful information that will improve treatment outcomes.

We recognize less than 200 tumors comprise the current analysis, but for a disease with an incidence of ~ 1 per million this is a large number that is likely very representative of the disease spectrum. The likelihood something important has been missed with these three analyses is small. Furthermore, although the totality of the data comes from three separate studies, strong concordance emerges, with a higher incidence of mutations in our cohort. The latter may reflect the more aggressive nature of the disease in our patients with aggressive metastatic presentations but given the lack of cancer-associated mutations, it most likely reflects a higher overall level of genomic instability—again with genomic integrity identified as “a pathway/biological process affected by associated consensus driver genes” in the TCGA analysis [30]. Furthermore, aggregating *ATRX*, *SETD2*, the mismatch repair and homologous recombination genes, and other genes involved in DNA repair, but excluding *TP53*, we find 36 mutations in samples obtained from 21 (49%) of the 43 patients suggesting impaired DNA repair may be an important theme in these aggressive ACCs an observation that could explain the higher incidence of mutations. However, we would again note that the mutations in ACC occurred in genes with longer transcript length, an observation that underscores the random nature of these mutations by suggesting a size driven and not function driven mutational profile.

## Conclusions

In conclusion, our data and published results suggest that the mutational profile in ACC does not explain carcinogenesis, biology, or resistance to therapy. Additionally, actionable mutations are not found in the large majority of ACCs. As observed with other cancers, DNA repair alterations may yet identify a subset sensitive to DNA damaging agents, and this needs to be explored more fully. Epigenetic alterations may represent a more fertile ground for investigation, particularly given the earlier reports demonstrating gene silencing via hypermethylation and its impact on prognosis. Much remains to be discovered about ACC.

## Supplementary information

**Additional file 1**. **Figure 1**: Plots compares targeted region coverage for 43 ACC tumors and the H295 ACC cell line (red dot) vs. the 25 matched normal samples (SureSelectXT v4 targeted regions). **A** Dot plots showing comparable read depth for normal and tumor samples (p = 0.73). Mean depth was 115 reads, normal (113 +/- 5.6, n = 25) and tumor (116 +/- 5.0, n = 44). **B** Line graph shows results for individual samples. **Figure 2**: Comparative copy number gains and losses. 17 of the 25 ACC tumors with paired normal DNA. The y-axis represents the percentage of samples with gain/loss. [ngCGH + Nexus segmentation (eight tumors removed due to excess noise)]. Qualitative comparison of the NCI data with the data of **A** Assie et al. [16] and Gara et al. [18] and with **B** Zheng et al. [8] **Figure 3**: Comparison of gene expression in tumors (ACC) and the normal adrenal (NA). Note wide range of expression for the ACC group. **A** Upregulated DEG **B** Downregulated DEG. There was a wide variation in the range of expression amongst the tumors. In the case of IGF2, for example, a gene whose expression has been previously reported to be high in ACCs both IGF2 high and low populations are seen as two different sub-groups. **Figure 4**: **A** Heat-map and 2-way unsupervised hierarchical clustering of our 57 ACC steroid-phenotype-low and –high and +/- proliferation samples based on the 136 K4 genes measured in our dataset. Eighteen samples, termed mixed, separated based on steroid phenotype but not on proliferation phenotype. All were C1A aggressive subtype. **B** Heat-map and 2-way unsupervised hierarchical clustering of the TCGA ACC samples based on 151 K4 genes (Table S2 in Zheng et al. [8]) that separate steroid-high from steroid-low (K4_2). **Figure 5**: Kaplan-Meier analysis of cDNA array data based on the K4 gene signature indicative of steroid phenotype low and high, or with (+) or without proliferation signature. As seen in the accompanying statistical analysis, the curves did not significantly differ in the samples derived from metastatic ACCs analyzed at the NIH. **Figure 6**: microRNA analysis, ACC vs. normal adrenal (NA). Figure shows 86 differentially expressed microRNA (DEM) with 17 upregulated, 69 downregulated DEMs (σ/σmax >0.2, p < 0.05, q = 0.10, >2-FC, one sample per patient, miRPlus probes removed). Four “normal adrenal-like” samples (8T, 16T, 36T and 49T) cluster with the normal adrenals but none of these correspond to the three “normal adrenal-like” samples based on mRNA profile.

**Additional file 2**. **Table 1**: (Part 1) List of Most Mutated Genes validated in >10% of 23 T/N pairs and >10% of all 43 ACC patients. (Part 2) List of Validated Driver Mutations in <10% of 43 ACC Patients. **Table 2**: A, Alterations in Chromatin Remodeling Genes or Genes Controlling Epigenetic Processes. B, Alterations in DNA Repair Genes. **Table 3**: 769 Differentially Expressed Genes (DEG), upregulated (289) and downregulated (480) in ACC compared to NA. **Table 4**: IPA prediction of upstream regulators based on microarray expression data comparing 57 ACC samples vs. 5 normal adrenal samples. **Table 5**: Top Differentially Expressed MicroRNAs (DEM), up- or down-regulated in ACC compared to NA.

## Data Availability

The datasets generated during the current study are available in the following repositories: Sequencing data have been deposited in NCBI Sequence Read Archives (SRA). *The link for the Fastq files in SRA:* https://trace.ncbi.nlm.nih.gov/Traces/study1/?acc=PRJNA596175. cDNA microarray and microRNA array data have been deposited in NCBI Gene Expression Omnibus (GEO). *The data deposited in GEO can be found under the following accession numbers: mRNA expression data*, GSE143383 https://www.ncbi.nlm.nih.gov/geo/query/acc.cgi?acc=GSE143383. *miRNA expression data*, GSE143385. https://www.ncbi.nlm.nih.gov/geo/query/acc.cgi?acc=GSE143385. Publicly available repositories used during study analysis and referred to in manuscript or in supplemental tables: COSMIC cancer gene census: https://cancer.sanger.ac.uk/census. GRCh37/hg19 genome assembly https://www.ncbi.nlm.nih.gov/assembly/GCF_000001405.13/. 1000 genomes https://www.internationalgenome.org/. NHLBI GO Exome Sequencing Project (ESP) https://evs.gs.washington.edu/EVS/. COMETE cancer atlas https://www.surrenales.com/reseaux-specialises-surrenales/institut-national-du-cancer-comete/ TCGA Pan Cancer Atlas https://www.cell.com/pb-assets/consortium/pancanceratlas/pancani3/index.html

## Abbreviations

ACC: Adrenocortical cancer
ATCC: American type culture collection
CA: California, USA
CCGC: COSMIC Cancer Gene Census
CML: Chronic myelogenous leukemia
CNV: Copy number variation
COMETE: French COMETE-Cancer network for adrenal cancer
COSMIC: Catalogue of Somatic Mutations in Cancer
GEO: Gene Expression Omnibus
CpG: Cytosine-phosphate-guanine dinucleotide
IRB: Institutional Review Board
MA: Massachusetts
MMR: Mismatch repair
NCI: National Cancer Institute
ngCGH: Comparative genomic hybridization
NIH: National Institutes of Health
NY: New York
PCR: Polymerase chain reaction
SRA: Sequence Read Archive
TCGA: The Cancer Genome Atlas
TMB: Tumor mutation burden
