# Supplementary material for: Metastatic and recurrent adrenocortical cancer is not defined by its genomic landscape
Source: BMC Med Genomics. 2020 Nov 4;13:165. doi: 10.1186/s12920-020-00809-7 (PMC7640690; doi:10.1186/s12920-020-00809-7)
Supplement: Supplementary file 1 — Additional file 1. Figure 1: Plots compares targeted region coverage for 43 ACC tumors and the H295 ACC cell line (red dot) vs. the 25 matched normal samples (SureSelectXT v4 targeted regions). A Dot plots showing comparable read depth for normal and tumor samples (p = 0.73). Mean depth was 115 reads, normal (113 +/- 5.6, n = 25) and tumor (116 +/- 5.0, n = 44). B Line graph shows results for individual samples. Figure 2: Comparative copy number gains and losses. 17 of the 25 ACC tumors with paired normal DNA. The y-axis represents the percentage of samples with gain/loss. [ngCGH + Nexus segmentation (eight tumors removed due to excess noise)]. Qualitative comparison of the NCI data with the data of A Assie et al. [16] and Gara et al. [18] and with B Zheng et al. [8] Figure 3: Comparison of gene expression in tumors (ACC) and the normal adrenal (NA). Note wide range of expression for the ACC group. A Upregulated DEG B Downregulated DEG. There was a wide variation in the range of expression amongst the tumors. In the case of IGF2, for example, a gene whose expression has been previously reported to be high in ACCs both IGF2 high and low populations are seen as two different sub-groups. Figure 4: A Heat-map and 2-way unsupervised hierarchical clustering of our 57 ACC steroid-phenotype-low and –high and +/- proliferation samples based on the 136 K4 genes measured in our dataset. Eighteen samples, termed mixed, separated based on steroid phenotype but not on proliferation phenotype. All were C1A aggressive subtype. B Heat-map and 2-way unsupervised hierarchical clustering of the TCGA ACC samples based on 151 K4 genes (Table S2 in Zheng et al. [8]) that separate steroid-high from steroid-low (K4_2). Figure 5: Kaplan-Meier analysis of cDNA array data based on the K4 gene signature indicative of steroid phenotype low and high, or with (+) or without proliferation signature. As seen in the accompanying statistical analysis, the curves did not significantly differ in the sam [file 12920_2020_809_MOESM1_ESM.pptx]

## Slide 1
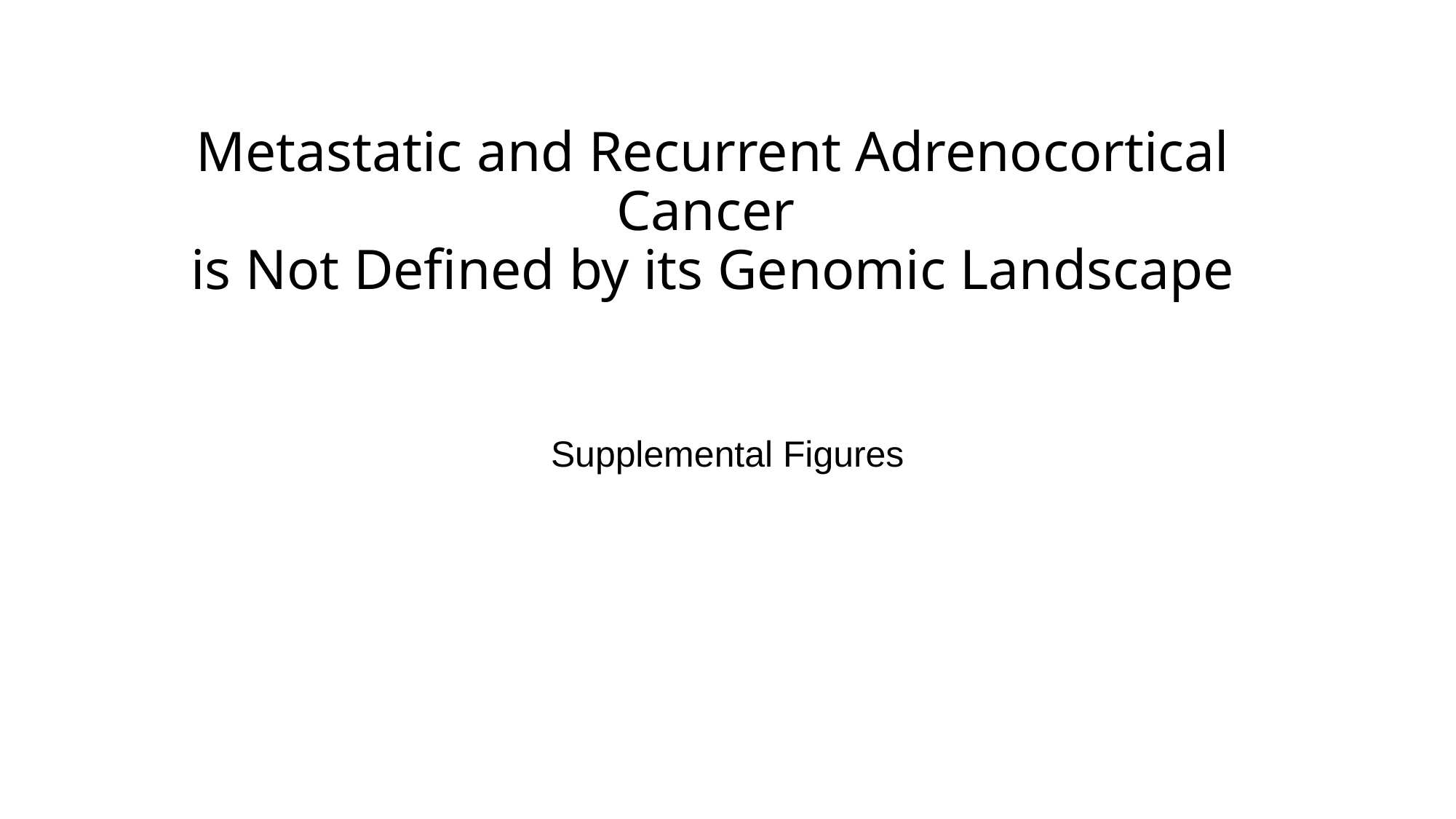

# Metastatic and Recurrent Adrenocortical Cancer is Not Defined by its Genomic Landscape
Supplemental Figures

## Slide 2
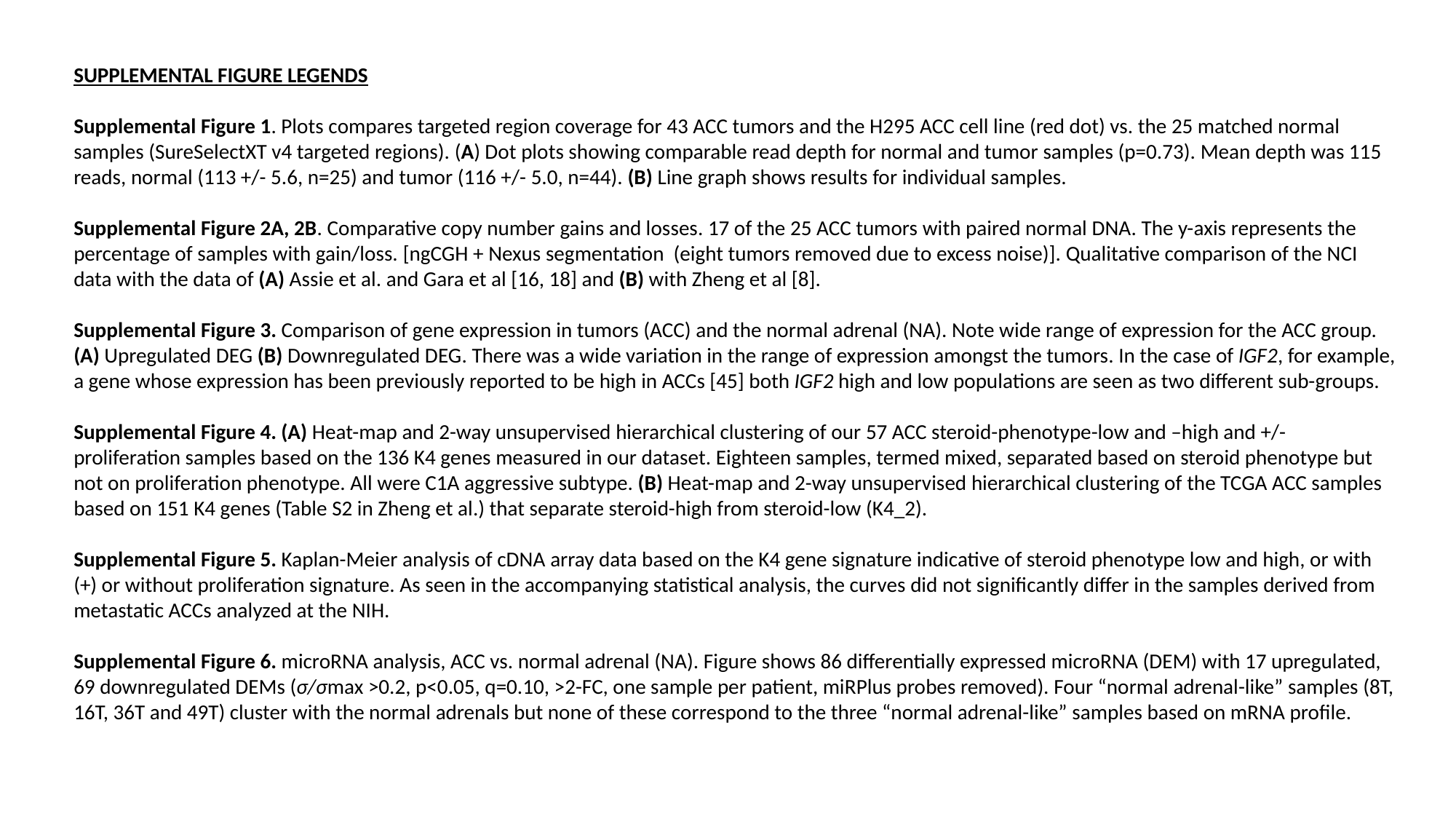

SUPPLEMENTAL FIGURE LEGENDS
Supplemental Figure 1. Plots compares targeted region coverage for 43 ACC tumors and the H295 ACC cell line (red dot) vs. the 25 matched normal samples (SureSelectXT v4 targeted regions). (A) Dot plots showing comparable read depth for normal and tumor samples (p=0.73). Mean depth was 115 reads, normal (113 +/- 5.6, n=25) and tumor (116 +/- 5.0, n=44). (B) Line graph shows results for individual samples.
Supplemental Figure 2A, 2B. Comparative copy number gains and losses. 17 of the 25 ACC tumors with paired normal DNA. The y-axis represents the percentage of samples with gain/loss. [ngCGH + Nexus segmentation (eight tumors removed due to excess noise)]. Qualitative comparison of the NCI data with the data of (A) Assie et al. and Gara et al [16, 18] and (B) with Zheng et al [8].
Supplemental Figure 3. Comparison of gene expression in tumors (ACC) and the normal adrenal (NA). Note wide range of expression for the ACC group. (A) Upregulated DEG (B) Downregulated DEG. There was a wide variation in the range of expression amongst the tumors. In the case of IGF2, for example, a gene whose expression has been previously reported to be high in ACCs [45] both IGF2 high and low populations are seen as two different sub-groups.
Supplemental Figure 4. (A) Heat-map and 2-way unsupervised hierarchical clustering of our 57 ACC steroid-phenotype-low and –high and +/- proliferation samples based on the 136 K4 genes measured in our dataset. Eighteen samples, termed mixed, separated based on steroid phenotype but not on proliferation phenotype. All were C1A aggressive subtype. (B) Heat-map and 2-way unsupervised hierarchical clustering of the TCGA ACC samples based on 151 K4 genes (Table S2 in Zheng et al.) that separate steroid-high from steroid-low (K4_2).
Supplemental Figure 5. Kaplan-Meier analysis of cDNA array data based on the K4 gene signature indicative of steroid phenotype low and high, or with (+) or without proliferation signature. As seen in the accompanying statistical analysis, the curves did not significantly differ in the samples derived from metastatic ACCs analyzed at the NIH.
Supplemental Figure 6. microRNA analysis, ACC vs. normal adrenal (NA). Figure shows 86 differentially expressed microRNA (DEM) with 17 upregulated, 69 downregulated DEMs (σ/σmax >0.2, p<0.05, q=0.10, >2-FC, one sample per patient, miRPlus probes removed). Four “normal adrenal-like” samples (8T, 16T, 36T and 49T) cluster with the normal adrenals but none of these correspond to the three “normal adrenal-like” samples based on mRNA profile.

## Slide 3
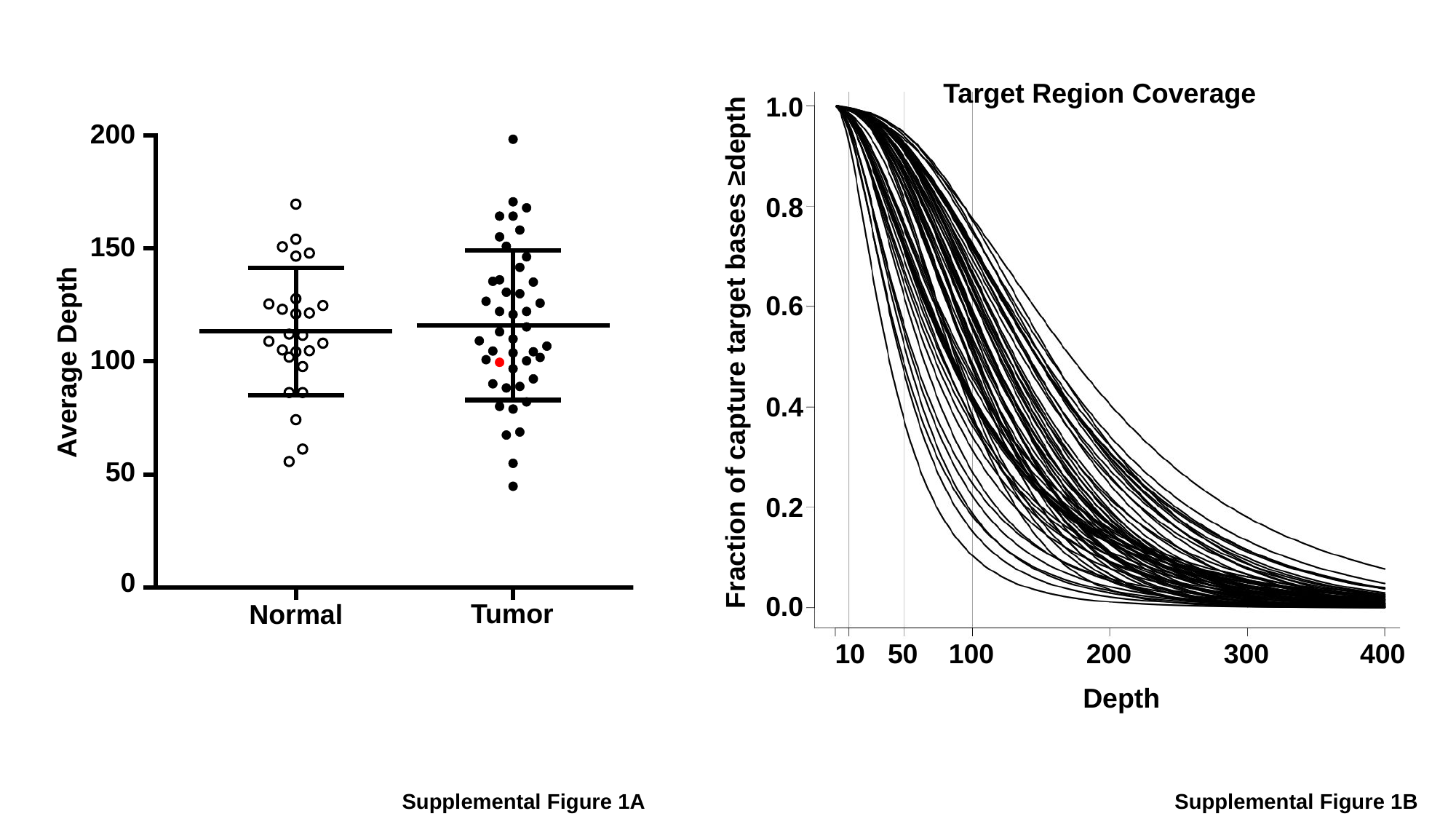

Target Region Coverage
1.0
0.8
0.6
0.4
0.2
0.0
Fraction of capture target bases ≥depth
10
50
100
200
300
400
Depth
Tumor
Normal
200
150
100
50
0
Average Depth
Supplemental Figure 1A
Supplemental Figure 1B

## Slide 4
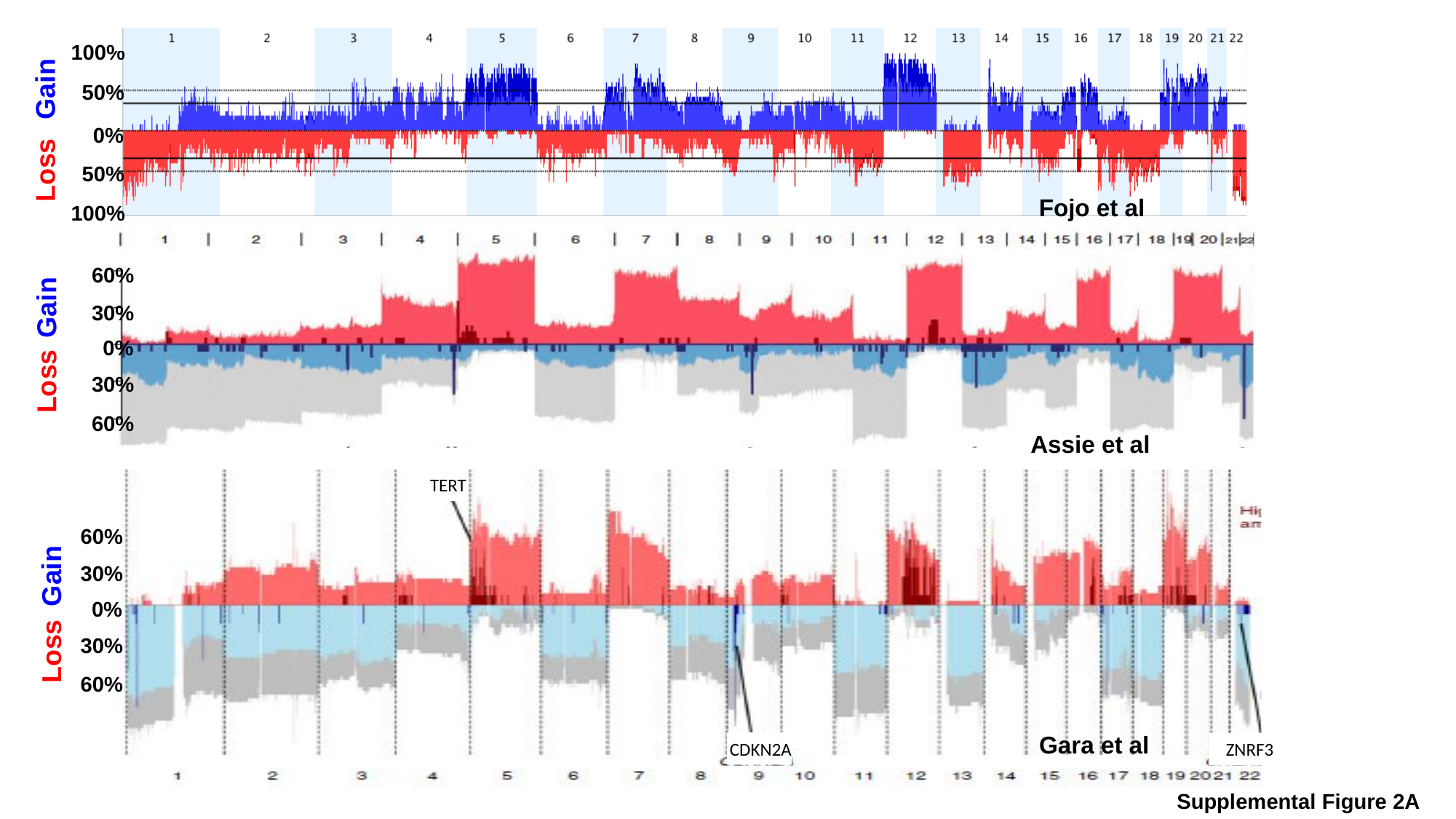

100%
50%
0%
50%
100%
Gain
Loss
Fojo et al
Gain
Loss
60%
30%
0%
30%
60%
Assie et al
TERT
60%
30%
0%
30%
60%
Gain
Loss
Gara et al
CDKN2A
ZNRF3
Supplemental Figure 2A

## Slide 5
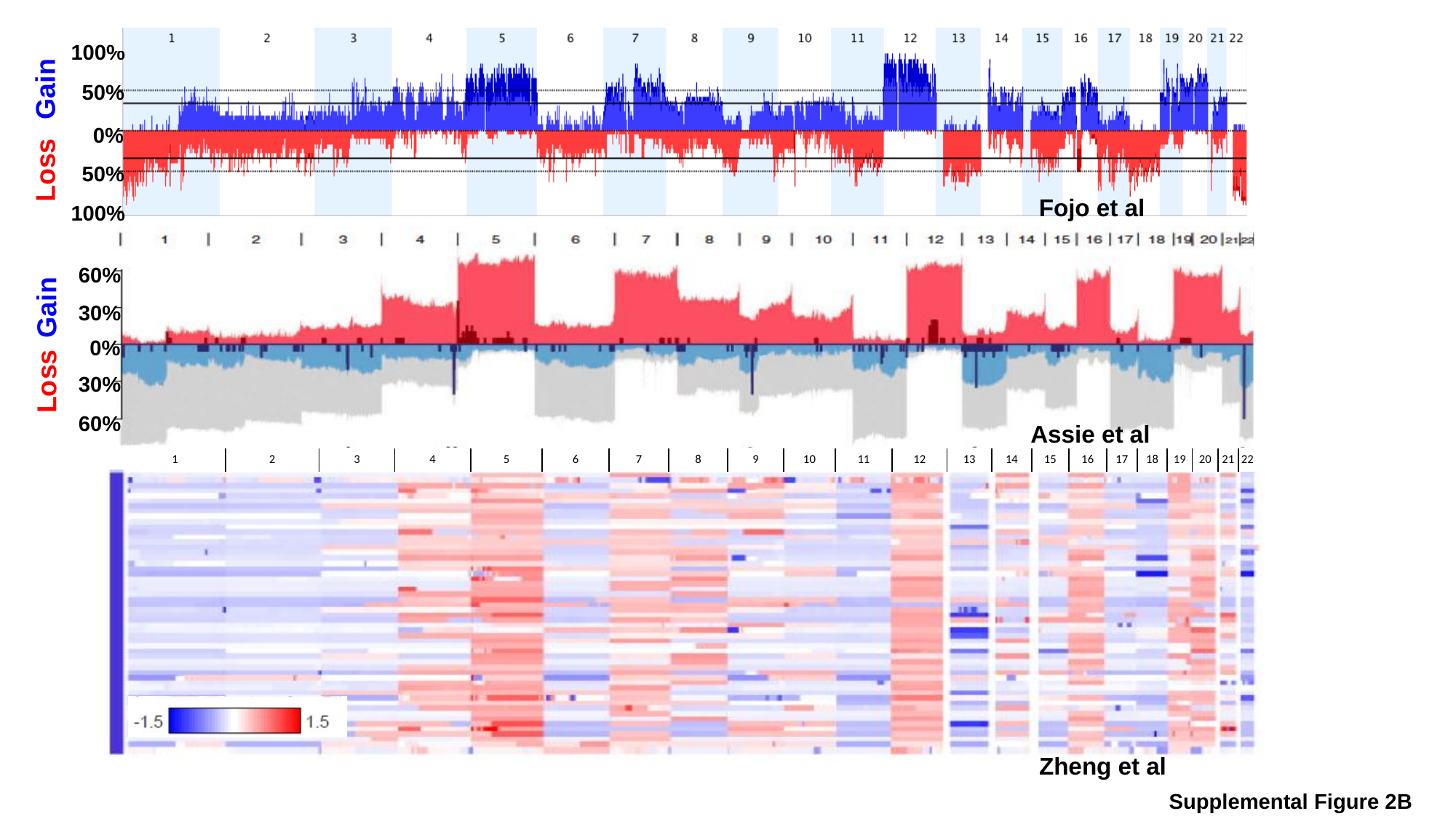

100%
50%
0%
50%
100%
Gain
Loss
Fojo et al
Gain
Loss
60%
30%
0%
30%
60%
Assie et al
| 1 | 2 | 3 | 4 | 5 | 6 | 7 | 8 | 9 | 10 | 11 | 12 | 13 | 14 | 15 | 16 | 17 | 18 | 19 | 20 | 21 | 22 |
| --- | --- | --- | --- | --- | --- | --- | --- | --- | --- | --- | --- | --- | --- | --- | --- | --- | --- | --- | --- | --- | --- |
Zheng et al
Supplemental Figure 2B

## Slide 6
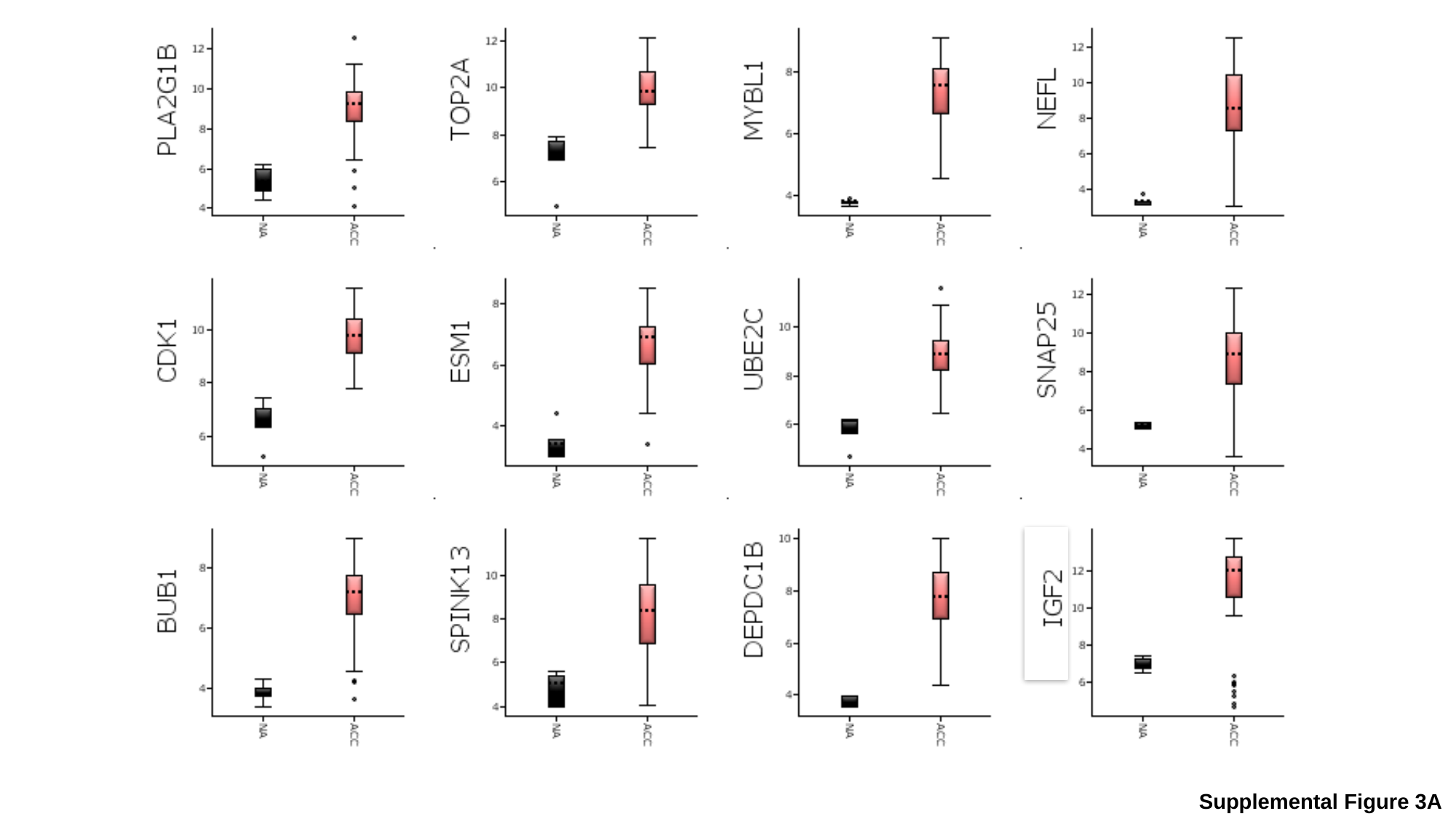

Supplemental Figure 3A

## Slide 7
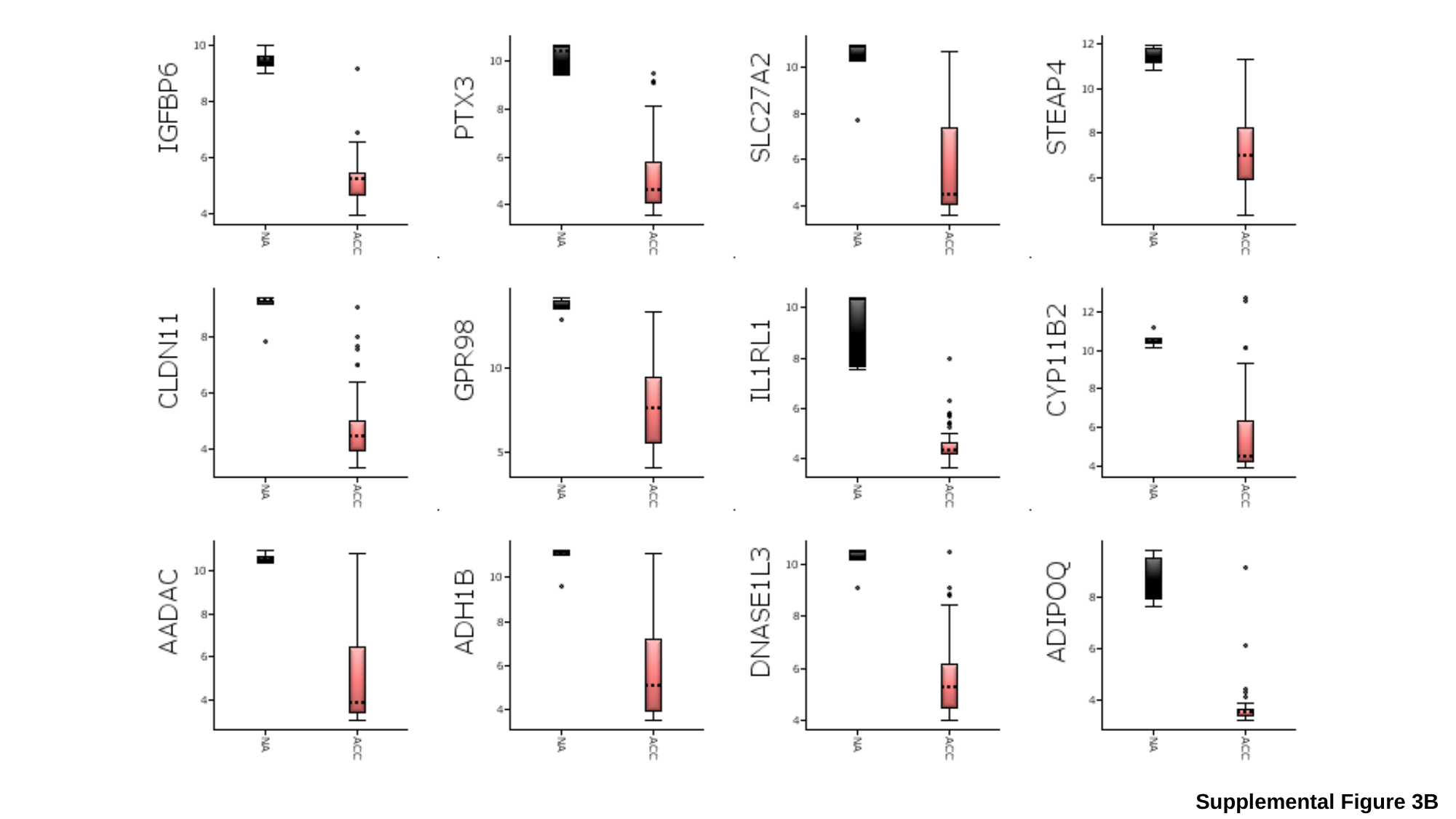

Supplemental Figure 3B

## Slide 8
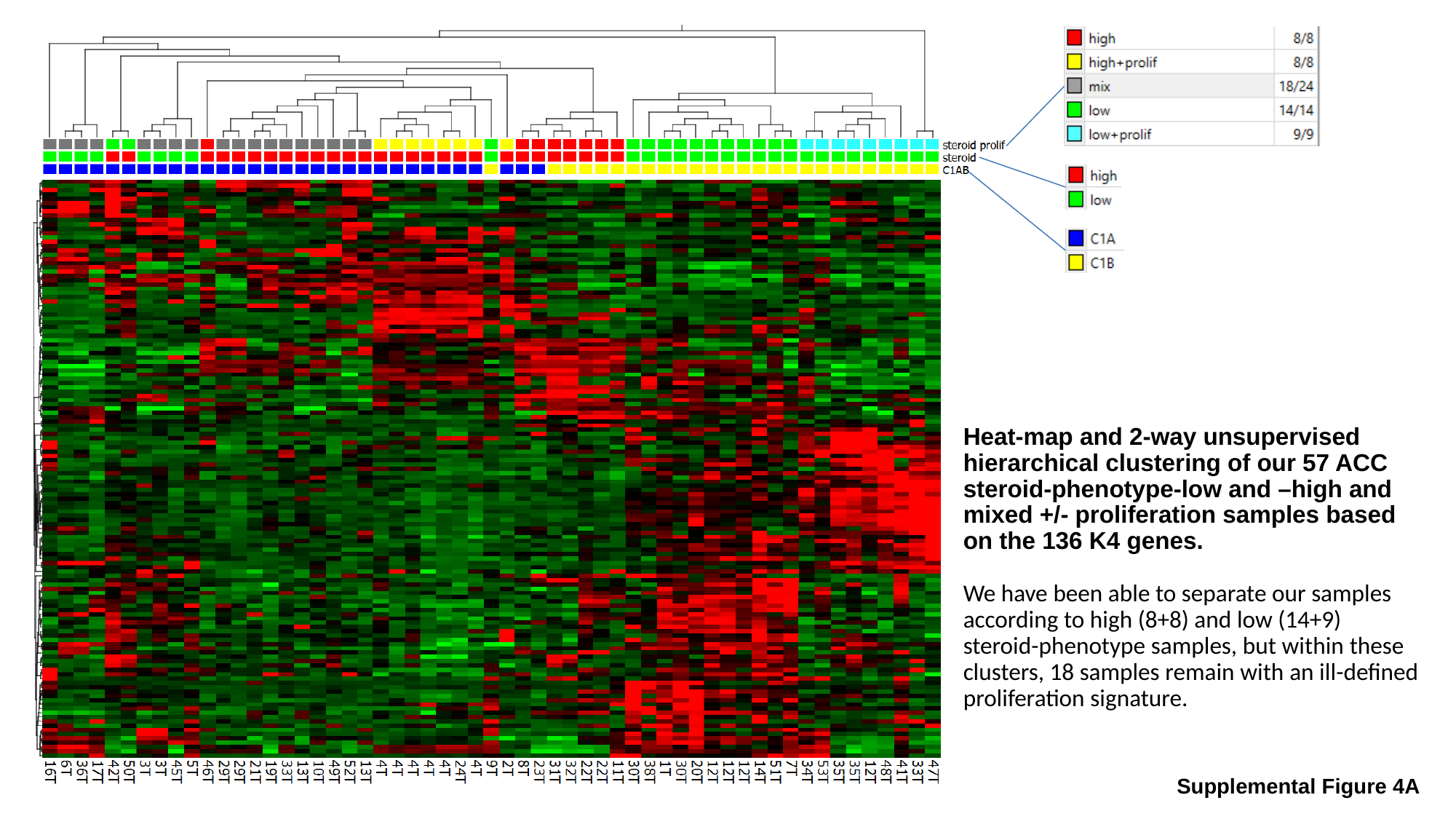

Heat-map and 2-way unsupervised hierarchical clustering of our 57 ACC steroid-phenotype-low and –high and mixed +/- proliferation samples based on the 136 K4 genes.
We have been able to separate our samples according to high (8+8) and low (14+9) steroid-phenotype samples, but within these clusters, 18 samples remain with an ill-defined proliferation signature.
Supplemental Figure 4A

## Slide 9
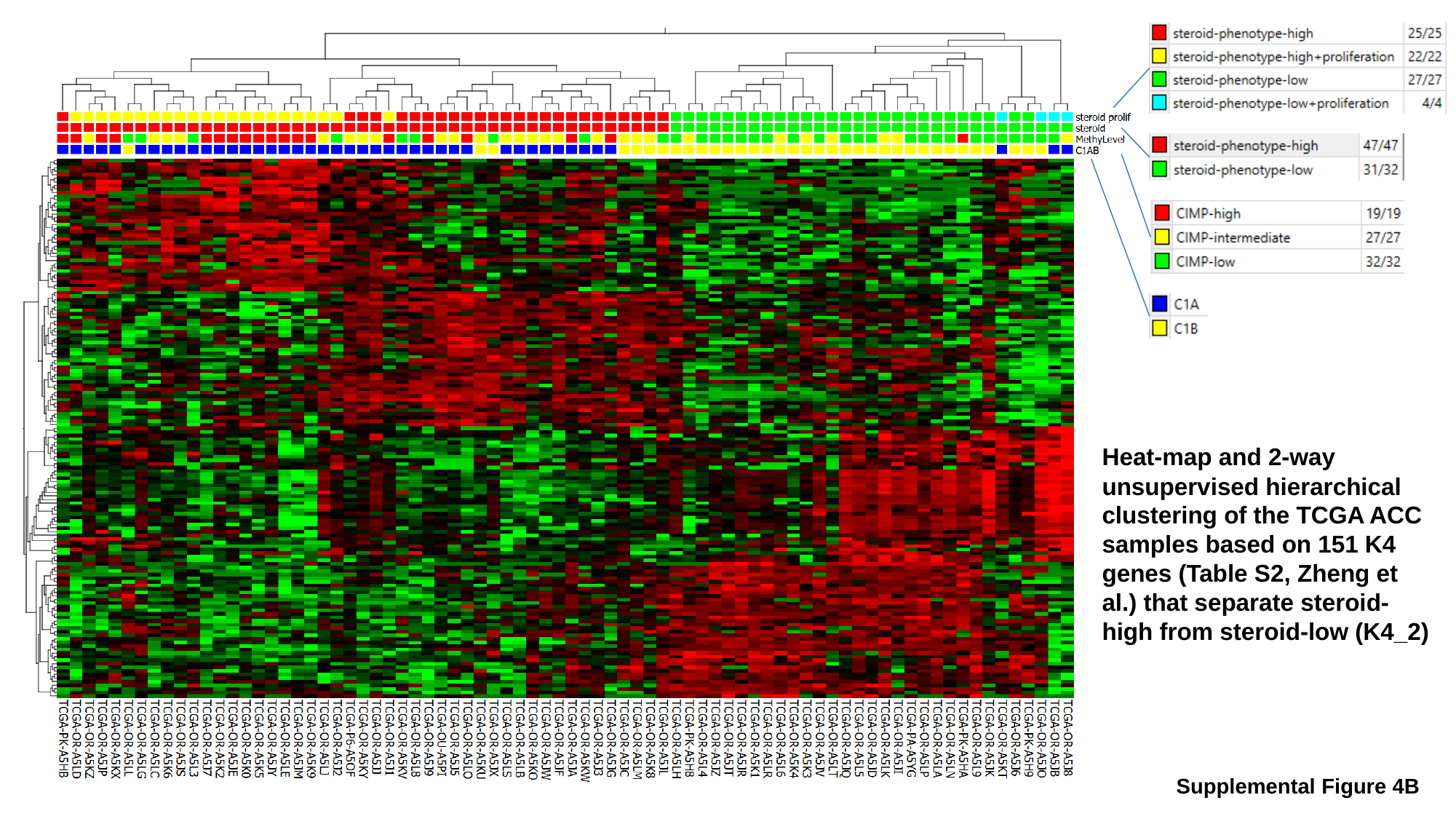

# Heat-map and 2-way unsupervised hierarchical clustering of the TCGA ACC samples based on 151 K4 genes (Table S2, Zheng et al.) that separate steroid-high from steroid-low (K4_2)
Supplemental Figure 4B

## Slide 10
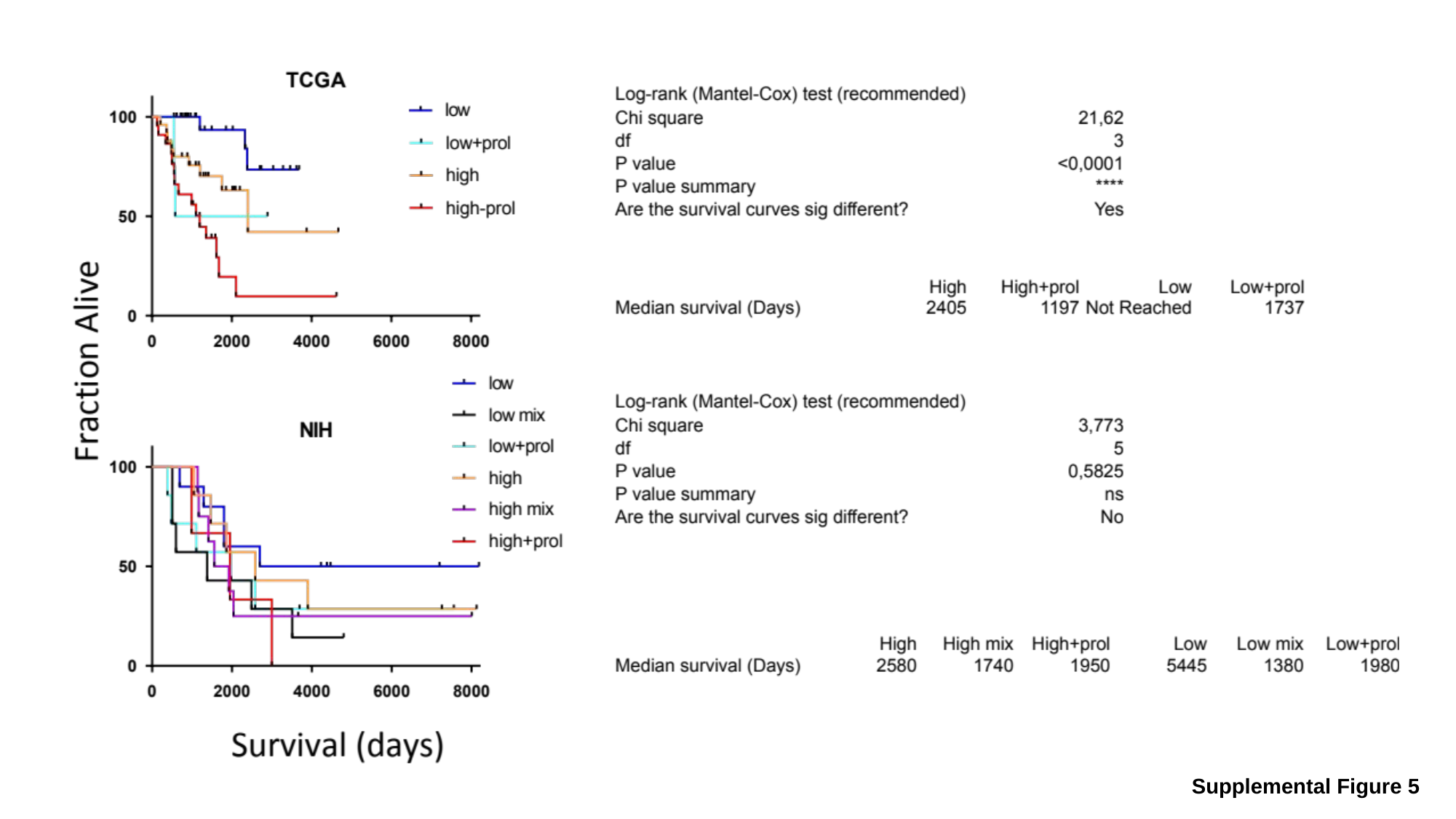

Supplemental Figure 5

## Slide 11
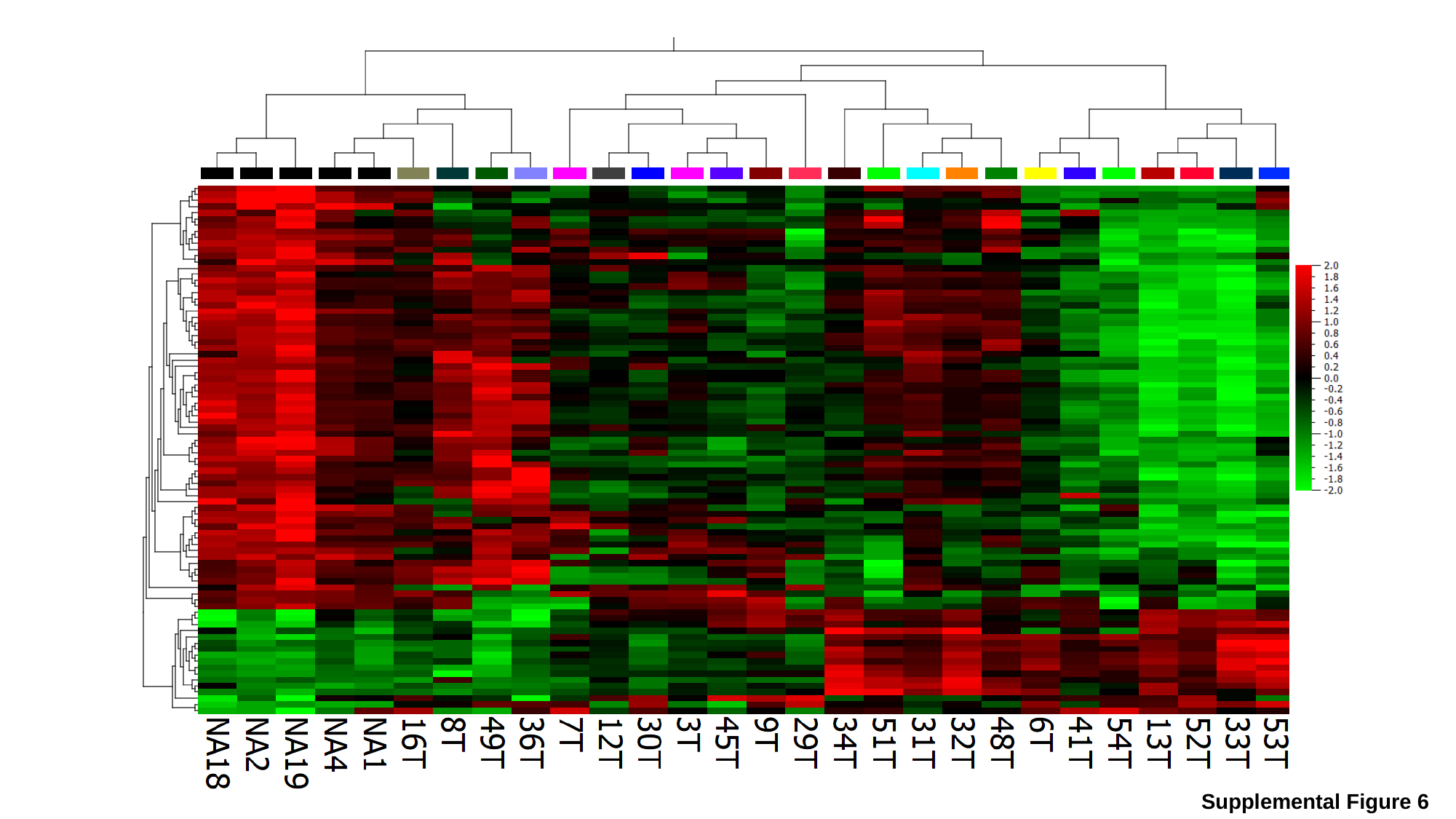

Supplemental Figure 6
